# Supplementary material for: Vitamin D Modified DSS-Induced Colitis in Mice via STING Signaling Pathway
Source: Biology (Basel). 2025 Jun 18;14(6):715. doi: 10.3390/biology14060715 (PMC12190092; doi:10.3390/biology14060715)
Supplement: Supplementary file 1 [file biology-14-00715-s001.zip › Supplementary Materials S1: 16s-report of gut microbiota/Groups/diff_analysis/Correlation/Genus-CAZY.pdf]

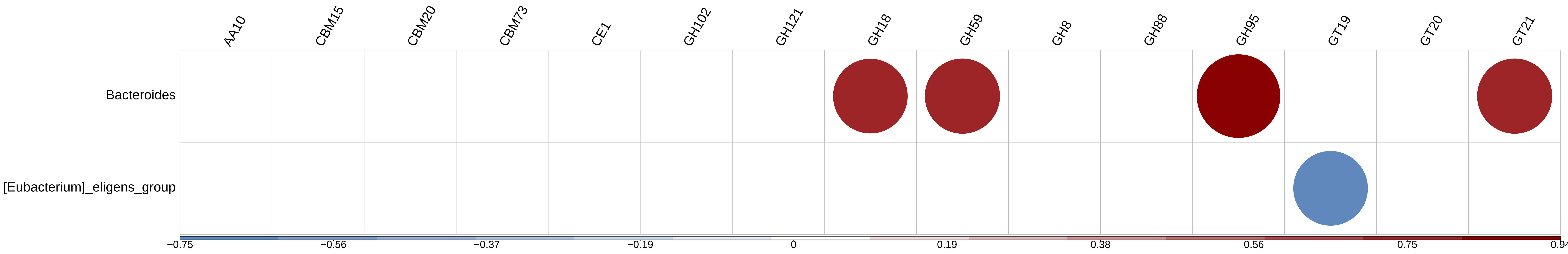

AA10
CBM15
CBM20
CBM73
CE1
GH102
GH121
GH18
GH59
GH8
GH88
GH95
GT19
GT20
GT21
Bacteroides
[Eubacterium]_eligens_group
−0.75
−0.56
−0.37
−0.19
0
0.19
0.38
0.56
0.75
0.94
